# Supplementary material for: Metabonomics and Transcriptomics Analyses Reveal the Development Process of the Auditory System in the Embryonic Development Period of the Small Yellow Croaker under Background Noise
Source: Int J Mol Sci. 2024 Feb 6;25(4):1954. doi: 10.3390/ijms25041954 (PMC10888356; doi:10.3390/ijms25041954)
Supplement: Supplementary file 1 [file ijms-25-01954-s001.zip › Supplementary material Figure S1 legend.pdf]

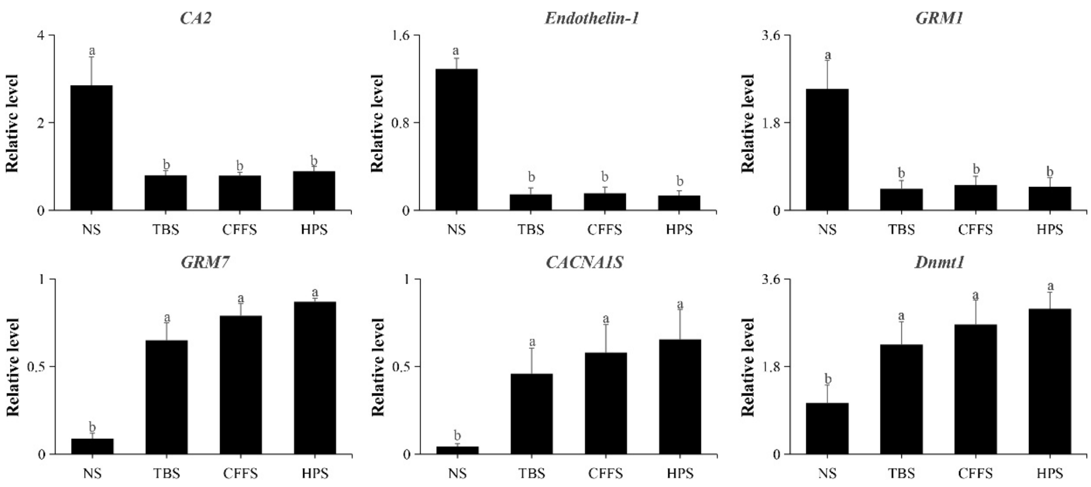

Supplementary material Figure S1. The results of qRT-PCR validation. The housekeeping gene was *GAPDH* (glyceraldehyde-3-phosphate dehydrogenase).
